# Clinically relevant antibiotic resistance in *Escherichia coli* from black kites in southwestern Siberia: a genetic and phenotypic investigation

**DOI:** 10.1128/msphere.00099-23

**Published:** 2023-06-13

**Authors:** Hassan Tarabai, Simon Krejci, Igor Karyakin, Ibrahim Bitar, Ivan Literak, Monika Dolejska

**Affiliations:** 1 Central European Institute of Technology (CEITEC), University of Veterinary Sciences, Brno, Czech Republic; 2 Department of Parasitology, Faculty of Science, University of South Bohemia, Ceske Budejovice, Czech Republic; 3 Department of Biology and Wildlife Diseases, Faculty of Veterinary Hygiene and Ecology, University of Veterinary Sciences, Brno, Czech Republic; 4 LLC Sibecocenter, Novosibirsk, Russia; 5 Biomedical Center, Charles University, Prague, Czech Republic; 6 Department of Clinical Microbiology and Immunology, Institute of Laboratory Medicine, The University Hospital, Brno, Czech Republic; Antimicrobial Development Specialists, LLC, Nyack, New York, USA

**Keywords:** *Milvus migrans*, *Escherichia coli*, ExPEC, APEC, wildlife, *qnrE1*, *mcr-1*, colistin resistance, IncI2, IncHI2

## Abstract

Wild birds including raptors can act as vectors of clinically relevant bacteria with antibiotic resistance. The aim of this study was to investigate the occurrence of antibiotic-resistant *Escherichia coli* in black kites (*Milvus migrans*) inhabiting localities in proximity to human-influenced environments in southwestern Siberia and investigate their virulence and plasmid contents. A total of 51 *E. coli* isolates mostly with multidrug resistance (MDR) profiles were obtained from cloacal swabs of 35 (64%, *n* = 55) kites. Genomic analyses of 36 whole genome sequenced *E. coli* isolates showed: (i) high prevalence and diversity of their antibiotic resistance genes (ARGs) and common association with ESBL/AmpC production (27/36, 75%), (ii) carriage of *mcr-1* for colistin resistance on IncI2 plasmids in kites residing in proximity of two large cities, (iii) frequent association with class one integrase (IntI1, 22/36, 61%), and (iv) presence of sequence types (STs) linked to avian-pathogenic (APEC) and extra-intestinal pathogenic *E. coli* (ExPEC). Notably, numerous isolates had significant virulence content. One *E. coli* with APEC-associated ST354 carried *qnrE1* encoding fluoroquinolone resistance on IncHI2-ST3 plasmid, the first detection of such a gene in *E. coli* from wildlife. Our results implicate black kites in southwestern Siberia as reservoirs for antibiotic-resistant *E. coli*. It also highlights the existing link between proximity of wildlife to human activities and their carriage of MDR bacteria including pathogenic STs with significant and clinically relevant antibiotic resistance determinants.

**IMPORTANCE:**

Migratory birds have the potential to acquire and disperse clinically relevant antibiotic-resistant bacteria (ARB) and their associated antibiotic resistance genes (ARGs) through vast geographical regions. The opportunistic feeding behavior associated with some raptors including black kites and the growing anthropogenic influence on their natural habitats increase the transmission risk of multidrug resistance (MDR) and pathogenic bacteria from human and agricultural sources into the environment and wildlife. Thus, monitoring studies investigating antibiotic resistance in raptors may provide essential data that facilitate understanding the fate and evolution of ARB and ARGs in the environment and possible health risks for humans and animals associated with the acquisition of these resistance determinants by wildlife.

## INTRODUCTION

Wild animals of various species are increasingly reported to host bacteria with diverse antibiotic resistance and virulence phenotypes including isolates with resistance to last-line antibiotics (1). Migratory birds have the potential to acquire and transmit antibiotic-resistant bacteria (ARB) and the associated resistance determinants in their breeding and wintering habitats and through their long migratory pathways (2). Previous reports have indicated raptors including black kites as significant carriers of Enterobacterales isolates resistant to clinically important antibiotics including last-line drugs such as carbapenems and colistin (3 - 5).

The black kite (*Milvus migrans*) is a common raptor species with an estimated population of 6 million individuals (6). The species include several subspecies that are distributed across Eurasia, Africa, and Australia (6, 7). Black kites have a unique ecological flexibility, which support their prosperity in various natural and human-influenced landscapes (8, 9). The black-eared kite (*Milvus migrans lineatus*) is a subspecies of black kites that inhabits Asia (10, 11) and recently, they were also reported across Western Europe (12). Black-eared kites breed in Asian temperate regions and migrate in winter to the Indian peninsula (11, 13, 14). Black kites have an opportunistic feeding behavior that utilizes food from various natural (small vertebrates including carrions) and anthropogenic sources (human food refuse from landfills) to meet their diet requirement (8, 11, 15, 16). All these factors involving black kite’s significant population, habitat, feeding habits, and long migration routes prime it to act as a model organism for investigating acquisition, prevalence, and transmission of ARB within avian wildlife.

*E. coli* with resistance to clinically important antibiotics were identified in human, environmental, domestic animals, and wildlife samples in Russia (5, 17, 18). A significant prevalence of *E. coli* resistant to quinolones and third-generation cephalosporins was reported in clinical and domestic animal samples from Russia (17, 19 - 21). In addition, several environment-focused studies have indicated Russian surface water and wastewater as a reservoir of ARB harboring resistance to a wide group of antibiotics including aminoglycosides, carbapenems, chloramphenicol, third-generation cephalosporins, and quinolones (18). However, studies investigating the carriage of ARB by Russian wildlife are scarce (5, 22). A recent study by our group indicated a black kite in Russian Siberia as a carrier of an *E. coli* isolate of sequence type (ST) 2280 harboring a mobile colistin resistance gene (*mcr-1*) that was located on conjugative IncI2 plasmid (5).

Here, we investigate the occurrence of antibiotic-resistant *E. coli* isolates from a large collection of Russian black kites (*n* = 55). The study utilized whole genome sequencing (WGS) data of 36 antibiotic-resistant *E. coli* from nestlings of black kites residing in three distinct habitats in the southwestern part of Russian Siberia. We investigated their phylogeny and carriage of antibiotic resistance genes (ARGs), virulence-associated genes (VAGs), and plasmid replicons and identified their sequence types (STs) and serotypes. Long read sequencing was utilized to investigate and characterize the plasmid content of one avian-pathogenic (APEC) *E. coli* isolate belonging to ST354.

## MATERIALS AND METHODS

### Sampling of black kites

**Fig 1:**
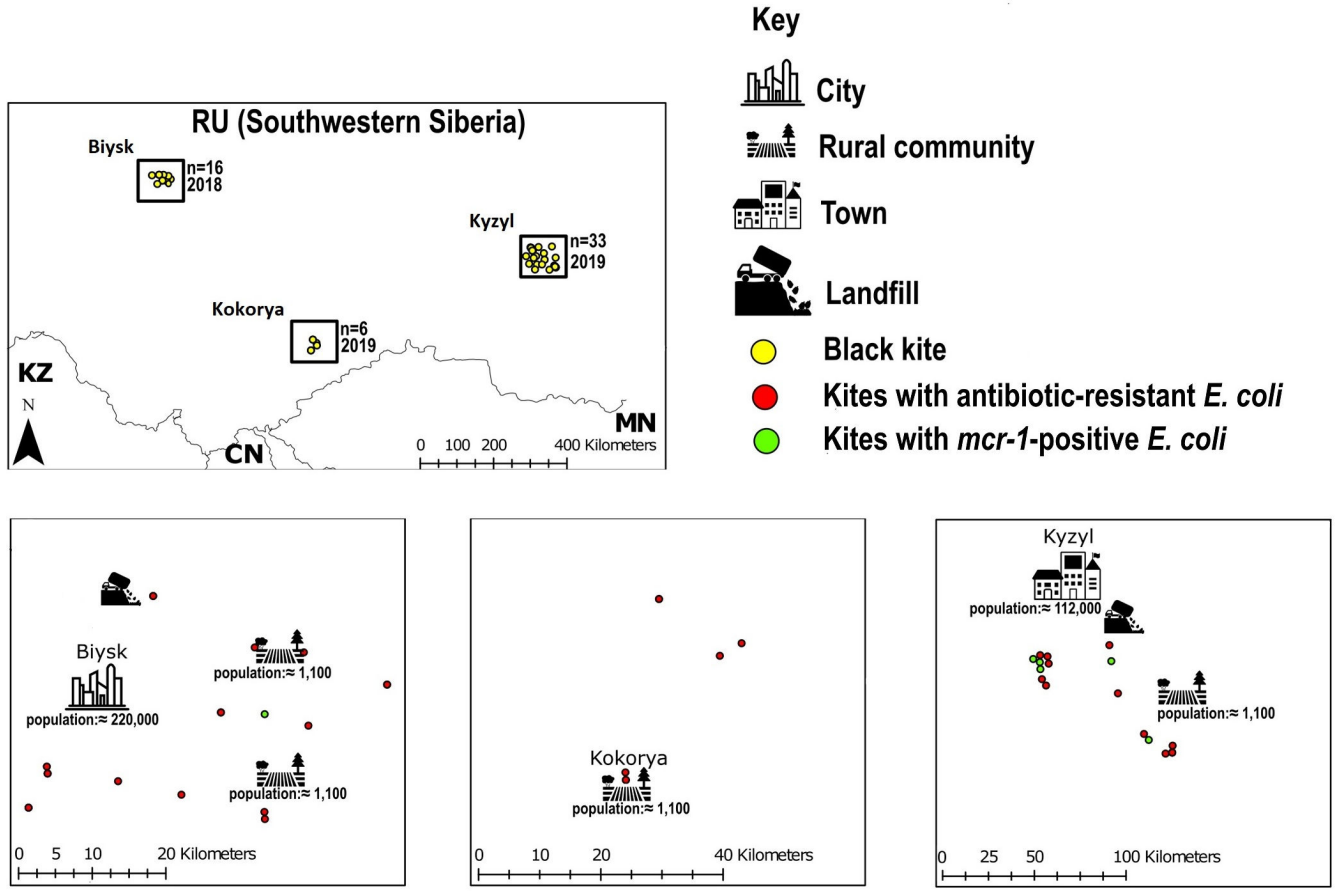
Schematic map showing the geographic distribution of black kites in southwestern Siberia including kites carrying antibiotic-resistant *E. coli* and *mcr-1*-positive isolates.

Cloacal samples (*n* = 55) from free-living nestlings of black kites were collected in their natural habitat through two consecutive years in 2018 (*n* = 16) and 2019 (*n* = 39) using swabs with culture medium (Amies transport medium with activated charcoal, Czech Republic), then transported to the laboratory and stored at 4°C till the initiation of the enrichment protocol. Sampling was performed in three localities in Russia’s southwestern Siberia (Fig. 1). Sampling localities included urban and agricultural areas around the cities of Biysk (*n* = 16, July 2018, Altai Krai) and Kyzyl (*n* = 33, June 2019, Republic of Tuva) and a small rural community in Kokorya (*n* = 6, June to August 2019, Altai Republic). (Refer to Table S1 for details on the sampling geolocation and collection year for each sample.)

### Selection of *E. coli* isolates

*E. coli* isolates were selected by cultivation of primary cloacal samples enriched overnight in peptone buffer (37°C with shacking at 140 RPM) on MacConkey agar with cefotaxime (2 mg/L), ciprofloxacin (0.05 mg/L), or meropenem (0.125 mg/L). In addition, SuperPolymyxin medium (23) was utilized for the selection of isolates with resistance to colistin. One presumptive *E. coli* isolates from each plate was taken and identified to species level by matrix-assisted laser desorption ionization time-of-flight mass spectrometry (24). Isolates identified as *E. coli* (*n* = 51) were subjected to further testing.

### Identification of antibiotic resistance genes

PCR was used to identify ARGs encoding resistance to beta-lactams (*bla*_ACC_ *, bla*_ACT_, *bla*_BIL_, *bla*_CMY_, *bla*_CTX-M_, *bla*_DHA_, *bla*_FOX_, *bla*_GES_, *bla*_OXA_, *bla*_LAT_, *bla*_MOX_, *bla*_SHV_, and *bla*_TEM_) (25), quinolones (*aac*(*6’*)*-Ib-cr*, *qnrA*, *qnrB*, *qnrC*, *qnrS*, *qnrD*, *qnrE*, *qepA*, *oqxA,* and *oqxB*) (26, 27), and colistin (*mcr-1-mcr-9*) (5, 28). *E. coli* isolates with at least one confirmed ARG (*n* = 36) were selected for WGS.

### Antibiotic susceptibility testing

Disc diffusion method was used to test the susceptibility of selected *E. coli* isolates from black kites to a set of 18 different antibiotics according to recommendations of the European Committee on Antimicrobial Susceptibility Testing (EUCAST) (29). The following antibiotic discs were used (Oxoid, Hants, UK): amoxicillin-clavulanic acid (20–10 µg), ampicillin (10 µg), azithromycin (15 µg), aztreonam (30 µg), cefazolin (30 µg), ceftazidime (30 µg), chloramphenicol (30 µg), ciprofloxacin (5 µg), ertapenem (10 µg), fosfomycin (200 µg), gentamicin (10 µg), imipenem (10 µg), nalidixic acid (30 µg), nitrofurantoin (300 µg) sulfonamide compounds (300 µg), streptomycin (10 µg), tetracycline (30 µg), and trimethoprim-sulfamethoxazole (1.25/23.75 µg). Measurement and interpretation of inhibition zone diameters of the tested isolates were based on EUCAST breakpoints (EUCAST 2019) or using breakpoints set by CLSI 2017 for antibiotics (azithromycin, cefazolin, tetracycline, nalidixic acid, sulfonamide compounds, and streptomycin) with no defined breakpoints in EUCAST 2019 (30, 31). EUCAST breakpoints are widely used in Europe, while CLSI breakpoints predominate in the USA. Both methodologies are recommended by World Health Organization; however, they lack harmonization with EUCAST breakpoints known to have higher resistance cut-offs and absence of intermediate resistance values or breakpoints for several antibiotics in *E. coli* and other bacterial genera compared to CLSI (32, 33). Susceptibility to colistin was assessed using colispot test (34) and interpreted based on EUCAST 2019 breakpoints (30). The production of AmpC beta-lactamase, extended-spectrum beta-lactamase (ESBL), and carbapenemase in *E. coli* isolates selected for WGS (*n*=36) was tested using AmpC, ESBL & Carbapenemase Set D72C (Mast Diagnostic, Merseyside, UK).

### Whole genome sequencing

Whole genome DNA was extracted from 36 *E. coli* isolates using NucleosSpin Microbial DNA kit (Macherey-Nagel, Germany). Preparation of DNA libraries was performed using Nextera XT DNA library preparation kit followed by sequencing on the NovaSeq platform (Illumina, San Diego, California, USA). Trimming of short reads for quality (Q ≤ 20) and adaptor residues was carried out using Trimmomatic v0.36 (35). Short reads were assembled using SPAdes v3.12.0 (36). Complete and closed plasmids of one *E. coli* isolates (DR162-CEF) belonging to APEC-linked ST354 were obtained using long-read sequencing. Whole genome DNA was extracted using a QIAGEN midi kit (Qiagen, Hilden, Germany), and library preparation was performed using microbial multiplexing based on the manufacturers’ recommendation. DNA was sheared using g-tubes (Covaris, Massachusetts, USA), but size selection was not performed for library preparation. Sequel 1 platform (Pacific Biosciences, California, USA) was used for sequencing followed by assembly using a Microbial Assembly pipeline in SMRT LNK v9.0 software (Pacific Biosciences, California, USA) with a minimum seed coverage of 30X. Quality control of obtained short- and long-read sequences was performed using FASTQC (https://www.bioinformatics.babraham.ac.uk/projects/fastqc/).

### *In silico* analysis of whole genome sequencing data

Publicly available tools were used for the analysis of sequenced *E. coli* isolates from black kites, which include FimTyper (v.1.0), SerotypeFinder (v.2.0), and MLST (v.2.0) (available at https://cge.cbs.dtu.dk/services/) to identify affiliation to serotype, *fimH* type, and ST, respectively. ARGs, VAGs, plasmid replicons, and their STs were determined using ResFinder (v.2.0) (available at https://cge.cbs.dtu.dk/services/), Virulence Factor Database (VFDB) (37), PlasmidFinder (v.2.0), and pMLST (v.2.0) (available at https://cge.cbs.dtu.dk/services/), respectively. Phylogroups of *E. coli* isolates were assigned using Clermont Typing (available at http://clermontyping.iame-research.center/). Automated annotation of complete and closed plasmids from DR162-CEF was performed using RASTtk (38) followed by manual curation by SnapGene (v.5.2.4, Biotech LLC, Chicago, USA) and BLASTn online tool (NCBI, Maryland, USA).

BRIG software (v.0.95) (39) and SnapGene (v.5.2.4, Biotech LLC, Chicago, USA) were used to perform a comparative analysis of plasmid sequences. A complete and closed *mcr-1*-positive-IncI2 plasmid (pDR164, GenBank accession no. MK542639) (5) originating from *E. coli* DR164-COL was used as a reference plasmid to investigate similar IncI2 plasmids within isolates from black kites’ populations. In addition, a comparative analysis of pDR164 with similar IncI2 plasmids from GenBank database was performed using BLASTn online tool (NCBI, Maryland, USA) with coverage thresholds of ≥99%. Closed and complete plasmids within *E. coli* DR162-CEF (pDR162-CEF-A, pDR162-CEF-B, and pDR162-CEF-C) were investigated for similar plasmids in GenBank with BLASTn using a coverage threshold of ≥88% for pDR162-CEF-A and ≥98% for pDR162-CEF-B and pDR162-CEF-C. A similar identity threshold of ≥99% was applied to all compared plasmids. BLASTn alignments were performed to identify class one integrase gene (*intI1*) and insertion sequence (IS) *26* elements.

### Phylogenetic analysis

Phylogenetic analysis based on single nucleotide polymorphisms (SNPs) was performed on all sequenced *E. coli* isolates from kites where *E. coli* K12-MG1655 was used as a reference genome. CSI Phylogeny 1.4 (available at https://cge.cbs.dtu.dk/services/) (40) was employed for SNPs analysis, and its results were visualized using iTOL (v.6.4) (41).

### *E. coli* metadata

Short read sequences of *E. coli* isolates from black kites were deposited on GenBank (BioProject ID PRJNA702622 ) and on EnteroBase in the *Escherichia*/*Shigella* database (Table S1 for accession and barcode numbers, respectively).

Complete and closed sequences of four plasmids from DR162-CEF isolate were deposited in GenBank (accession nos. MW651977, MW651978, MW651979, and MW651980).

### Transferability of quinolone and beta-lactam resistance

A conjugation assay based on filter mating method was performed on *qnrE1*-positive IncHI2-ST3 (pDR162-CEF-A) and *bla*_CMY-2_-positive IncI1-Iα (pDR162-CEF-B) plasmids. The plasmid-free, rifampicin- and azide-resistant *E. coli* MT102 (42) was used as a recipient strain for the conjugative transfer of pDR162-CEF-A and pDR162-CEF-B. Incubation of mating strains was performed on Luri (LB) agar plates at 28°C for pDR162-CEF-A and 37°C for pDR162-CEF-B for 4 h. This was followed by selection of transconjugants on LB plates with ciprofloxacin (0.05 mg/L), rifampicin (25 mg/L), and sodium azide (100 mg/L) for pDR162-CEF-A and cefotaxime (2 mg/L), rifampicin (25 mg/mL), and sodium azide (100 mg/L) for pDR162-CEF-B with incubation overnight at 37°C. The presence of *qnrE1*, *bla*_CMY-2_, IncHI2, and IncI plasmids in the transconjugants was confirmed by PCR (26, 43) and replicon typing (44). Transferability of *mcr-1*-positive IncI2 plasmid in one isolate (DR164-COL) was confirmed previously (5).

## RESULTS

### Carriage of multidrug-resistant *E. coli* is common among black kites

*E. coli* isolates (*n* = 51) with reduced susceptibility to clinically important antibiotics including cefotaxime (22/51, 43%), ciprofloxacin (22/51, 13%), and colistin (7/51, 5%) were recovered from cloacal samples of black kites (35/55, 63.6%). Notably, isolates with resistance to meropenem were absent from all kites. Most (48/51, 94.1%) isolates expressed MDR profiles with phenotypic resistance to three or more classes of antibiotics. ESBL/AmpC production was identified in the majority of sequenced isolates (27/36, 75%). Resistance to aminoglycosides, beta-lactams, fluoroquinolones, macrolides, phenicol, tetracycline, and trimethoprim was observed in numerous *E. coli* isolates, while isolates with colistin resistance phenotype (7/36, 19.4%) were identified in black kites residing in proximity to Biysk (1/16, 6%) and Kyzyl (6/33, 18%) cities. Fosfomycin resistance was only observed in isolates from samples collected around Biysk city (1/16, 6%) (Table S1).

#### Genomic profiles and phylogenetic analysis highlight high diversity of *E. coli*

WGS revealed high diversity of *E. coli* isolates in terms of their phylogroups, STs, serotypes, and *fimH* profiles. They were associated with all seven *E. coli* phylogroups (A, B1, B2, C, D, E, and F) with a predominance of phylogroups B1 (14/36, 38%) and A (11/36, 30%). A total of 30 different STs including four novel STs were observed, most of which were represented by single *E. coli* isolates while ST162 was the most common ST (4/36, 11.1%). Common *fimH* types included *fimH32* (10/36, 27%) and *fimH31* (4/36, 11%) (Table S1).

**Fig 2:**
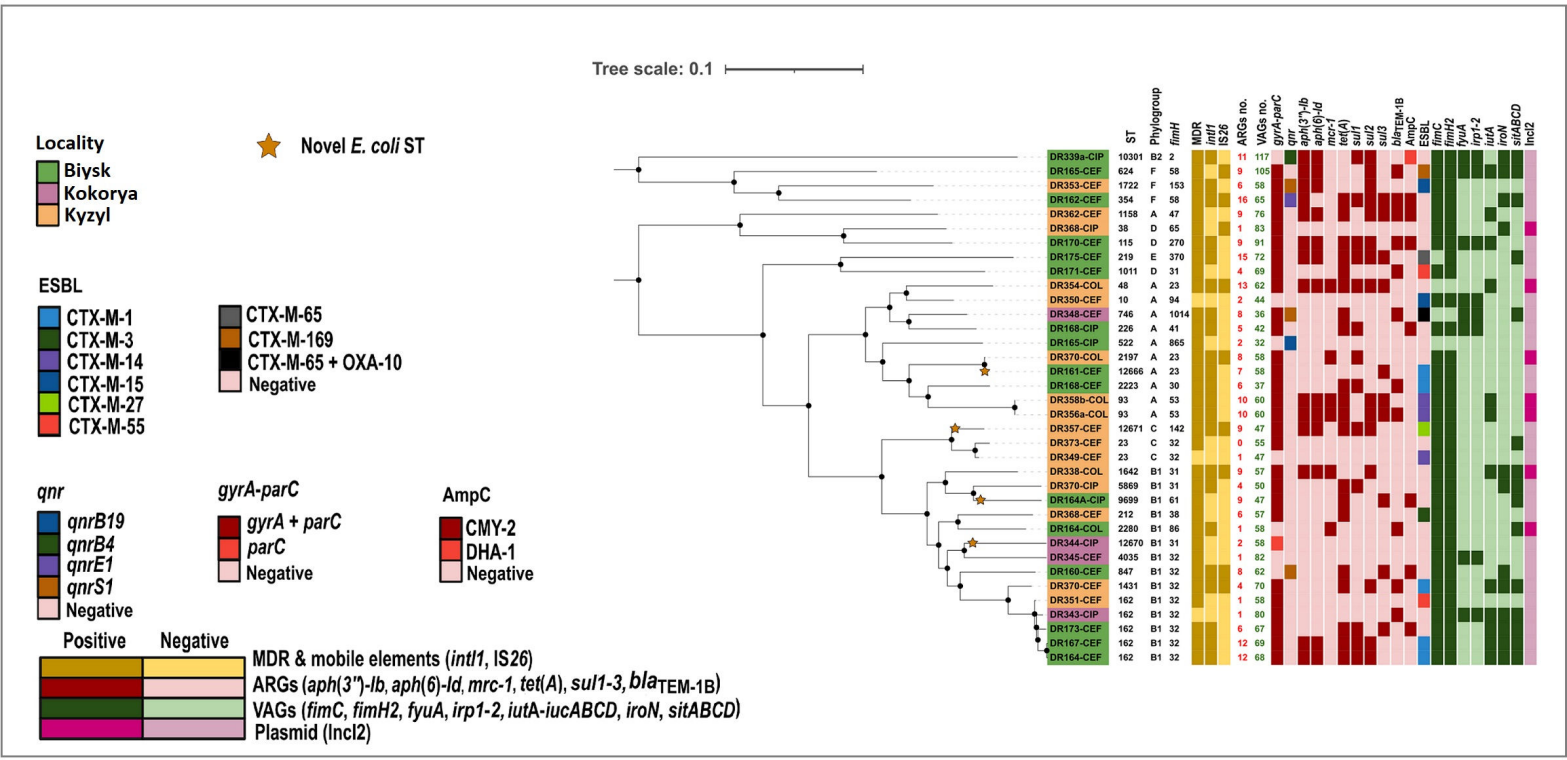
Clonal relationship of *E. coli* isolates (*n* = 36) from black kites (*n* = 55) in Biysk (*n* = 16), Kokorya (*n* = 6), and Kyzyl (*n* = 33).

Phylogenetically, isolates from black kites had high heterogeneity (0–46714 SNP variants) with no observed clustering (Fig. 2). Two *E. coli* ST162 isolates with MDR and ESBL phenotypes from kites in Biysk region (DR164-CEF and DR167-CEF) were closely related (10 SNPs difference) and shared identical *fimH* type (32), serotype (O88:H10), and ARGs content. Similarly, two isolates originating from kites in proximity to Kyzyl city had two clonal *mcr-1*-positive isolates (DR356a-COL, and DR358b-COL with 0 SNPs difference) belonging to ST93 with *fimH*53 and serotype O21:H16. Both of the latter isolates were ESBL producers and shared identical ARGs and VAGs content and a highly similar plasmid profile. Two isolates from kites in proximity to Biysk and Kyzyl cities (DR161-CEF ST2197 and DR370-CEF ST12666, respectively) were closely aligned (103 SNPs difference) and had identical *fimH* type (23) and serotype (O128:H26) as well as virulence profile. However, their ARGs and plasmid content were distinct with the exception of sharing *tet*(*B*) and IncI1-I plasmid.

#### *E. coli* from black kites bear a notable pool of antibiotic resistance genes

Sequenced isolates carried between 0 and 16 ARGs with an average of 7 ARGs acting on 10 different classes of antibiotics. Most common ARGs included *tet*(*A*) (20/36, 55%), *sul2* (15/36, 41%), *aph*(*3''*)*-Ib* (14/36, 38%), *aph* (6)*-Id*, *sul1* (13/36, 36%, respectively), *bla*_TEM-1B_ (11/36, 30%), and *sul3* (10/36, 27%). All of the latter ARGs except for *tet*(*A*) and *bla*_TEM-1B_ were absent from isolates originating from black kites in Kokorya rural community.

**Fig 3:**
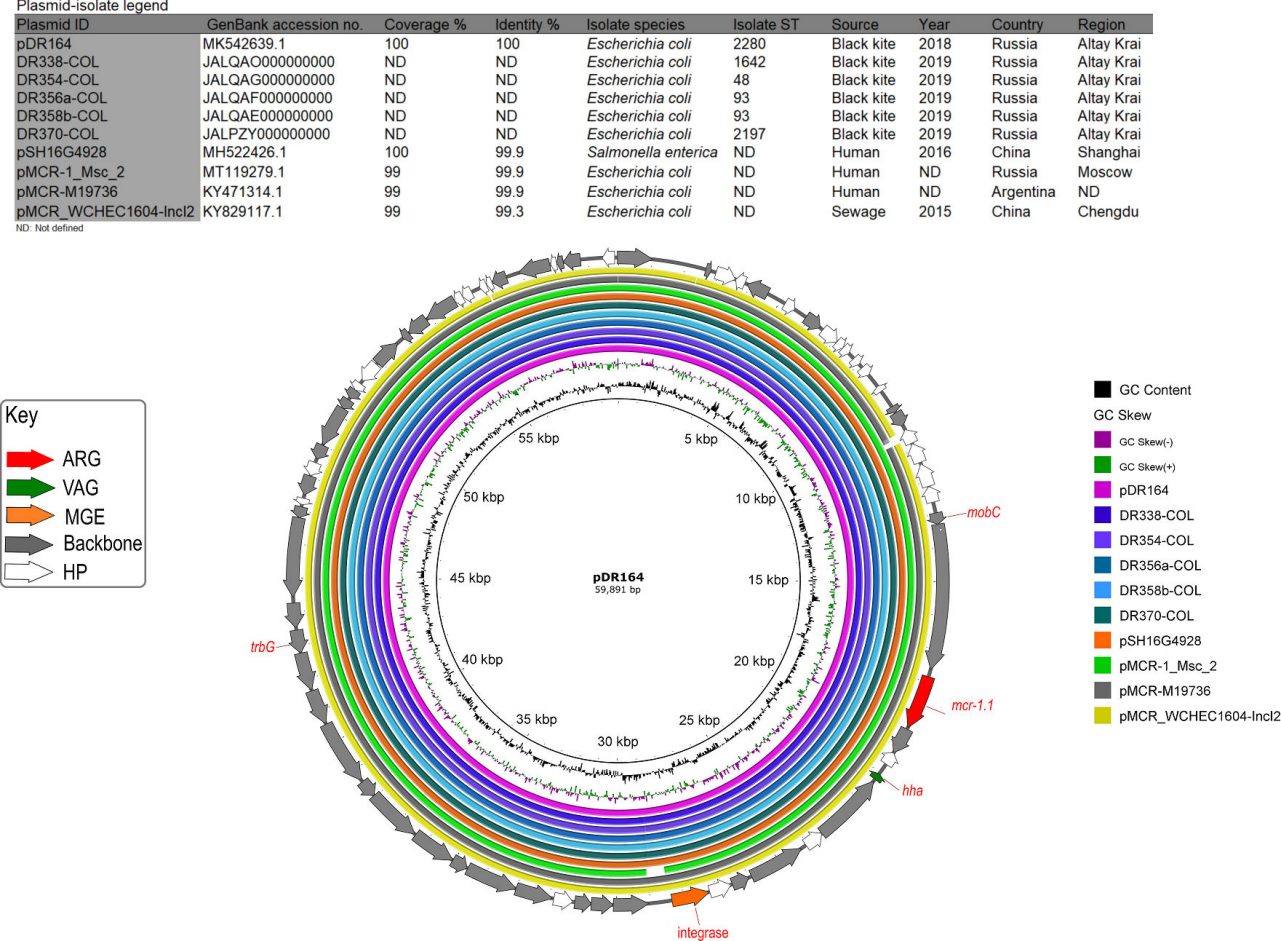
BRIG comparison of *mcr-1*-positive IncI2 plasmid pDR164 with similar plasmid sequences retrieved from isolates of black kites in Kyzyl (*n* = 5) and from GenBank. In the key, ARG: antibiotic resistance gene, VAG: virulence-associated gene, MGE: mobile genetic element, and HP: hypothetical protein.

Isolates with colistin resistance phenotype were identified in black kites residing around Biysk (1/16, 6.2%) and Kyzyl (6/33, 18.1%) cities, all harboring *mcr-1-*positive IncI2 plasmids (Fig. 3). Most sequenced isolates carried ESBL and AmpC genes (18/36, 50% and 9/36, 25%, respectively) but isolates from Biysk lacked acquired AmpC genes. Eight variants of *bla*_CTX-M_ gene were identified in the sequenced isolates with *bla*_CTX-M-1_ (5/36, 13.8%) as the most common variant (Table S1). Among plasmid-mediated quinolone resistance (PMQR) genes, *qnrS1* was observed in three isolates of different STs originating from kites in Biysk, Kyzyl, and Kokorya (3/36, 8.3%). Other identified PMQR genes included *qnrB19* and *qnrE1* (2/36, 5.5%, respectively) that were exclusively present in individual samples from Biysk (1/15, 6%, respectively) and *qnrB4* (1/36, 2.7%) carried by an isolate originating from a kite in Kyzyl (1/17, 5%). Chromosomal mutations in *gyrA* (DNA gyrase subunit A) and *parC* (topoisomerase IV subunit) genes conferring resistance to fluoroquinolones were frequently found among sequenced isolates (29/36, 80.5% and 28/36, 77.7%, respectively) (Table S1).

#### Significant carriage of virulence-associated genes among black kite *E. coli* isolates

Several isolates (9/36, 25%) belonged to ExPEC and APEC-linked STs including ST10, ST23, ST38, ST93, ST354, ST624, and ST1011 and were distributed among kites from Biysk, Kyzyl, and Kokorya. Isolates carried 32 to 117 VAGs with an average of 63. Various isolates harbored genes associated with adhesion *fimH2* (35/36, 97%), *fimC* (32/36, 88%), and *papC* (3/36, 8%); siderophores *iroN*, *iutA, iucABCD* (12/36, 33%, respectively), *fyuA* (8/36, 22%) and *irp1-2* (8/36, 22%); and ferrous acquisition system *sitABCD* (15/36, 41%). Three isolates from kites in Biysk (DR344-CIP, ST12670) and Kyzyl (DR349-CEF and DR373-CEF, ST32, respectively) carried the *rhs*/PAAR toxin gene. One isolate (DR339a-CIP) with the highest number of virulence genes (*n* = 117) was sourced from a kite in Kyzyl and belonged to ST10301. Its virulence profile included several VAGs that were not identified in other isolates. This included genes encoding invasins (*afaD* and *afaE),* colibactin operon (*colABCDEFGHIJKLMNPQS*), toxins (*cnf1* for cytotoxic necrotizing factor type 1, *hylA* for α-hemolysin, *vat* encoding vacuolating autotransporter toxin, and *senB* for enterotoxin TieB), iron acquisition system (*chuA* for hemin receptor molecule), and a homologous Toll/interleukin-1 receptor (*tcpC*) (Table S1).

#### High diversity of plasmid replicons and spread of *intI1* and IS*26* in *E. coli* from black kites

In total, 18 different plasmid replicons were identified with *F* and various Col plasmids present in most isolates (28/36, 77% and 27/36, 75%, respectively). Sequenced isolates carried between zero and nine plasmids with an average of three plasmids (Table S1).

Corresponding replicon sequence types (RSTs) of all *F* plasmids were present in single isolates with several exceptions. F18:A-:B1 plasmid was identified in three isolates belonging to kites from Biysk, Kyzyl, and Kokorya. Two of these isolates (DR173-CEF from Biysk and DR343-CIP from Kokorya) shared a common ST162, while the isolate from Kyzyl (DR338-COL) was of ST1642. IncI-I plasmids were present in 11/36 (30.5%) isolates and belonged to clonal complex (CC) 2 and CC3. They belonged to six different STs with ST3 predominating (5/11, 45.4%). Two isolates carried HI2 plasmids, one belonged to ST3 carrying *qnrE1* gene (DR162-CEF) while another had a nontypeable ST with close relation to ST3. (Refer to Table S1 for characterization and distribution of replicon STs.)

Class one integrase gene *intI1* was common among the isolates (22/36, 61.1%) and present in *E. coli* from Biysk (*n* = 11), Kyzyl (*n* = 10), and Kokorya (*n* = 1). IS*26* (9/36, 25%) was only identified in isolates from kites in Kyzyl (*n* = 6) and Biysk (*n* = 3) (Fig. 2).

#### Resistance to colistin associated with *mcr-1-*IncI2 plasmids

The *mcr-1* gene in all six isolates from kites in Biysk and Kyzyl was carried on IncI2 plasmids with highly homologous structures (Fig. 3). Isolates harboring *mcr-1* were MDR, belonged to diverse STs (48, 93, 1,642, 2,197, and 2,280), phylogroups A (4/6) and B1 (2/6), and three of them were ESBL producers. Comparative analysis of IncI2 plasmids from black kites (Fig. 3) showed high identity and coverage with IncI2 plasmids originating from clinical samples in China (hosted by *Salmonella enterica*), Argentina, and Russia (hosted by *E. coli*) and a sewage sample in China (harbored in *E. coli*).

#### Plasmids of *E. coli* DR162-CEF carry genes for MDR, metal resistance, and colicin production

Isolate DR162-CEF originated from a black kite in Biysk was MDR and AmpC producer and belonged to ST354 and phylogroup *F* with *fimH*58 and serotype O1:HNT. Notably, in addition to plasmid-mediated resistance, DR162-CEF encoded chromosomal resistance to aminoglycosides (*aac* (3)*-Iva*, *aph*(*3"*)*-Ib*, *aph* (4)*-Ia*, and *aph* (6)*-Id*), phenicol (*cmlA*), sulfonamides (*sul1*, *sul2*, and *sul3*), tetracycline [*tet*(31), *tet*(A), and *tet*(M)] and trimethoprim (*dfrA7*). Its virulence content included genes associated with pathogenic *E. coli* including *fimH2*, *fimC, iroN,* and *sitABCD*.

**Fig 4:**
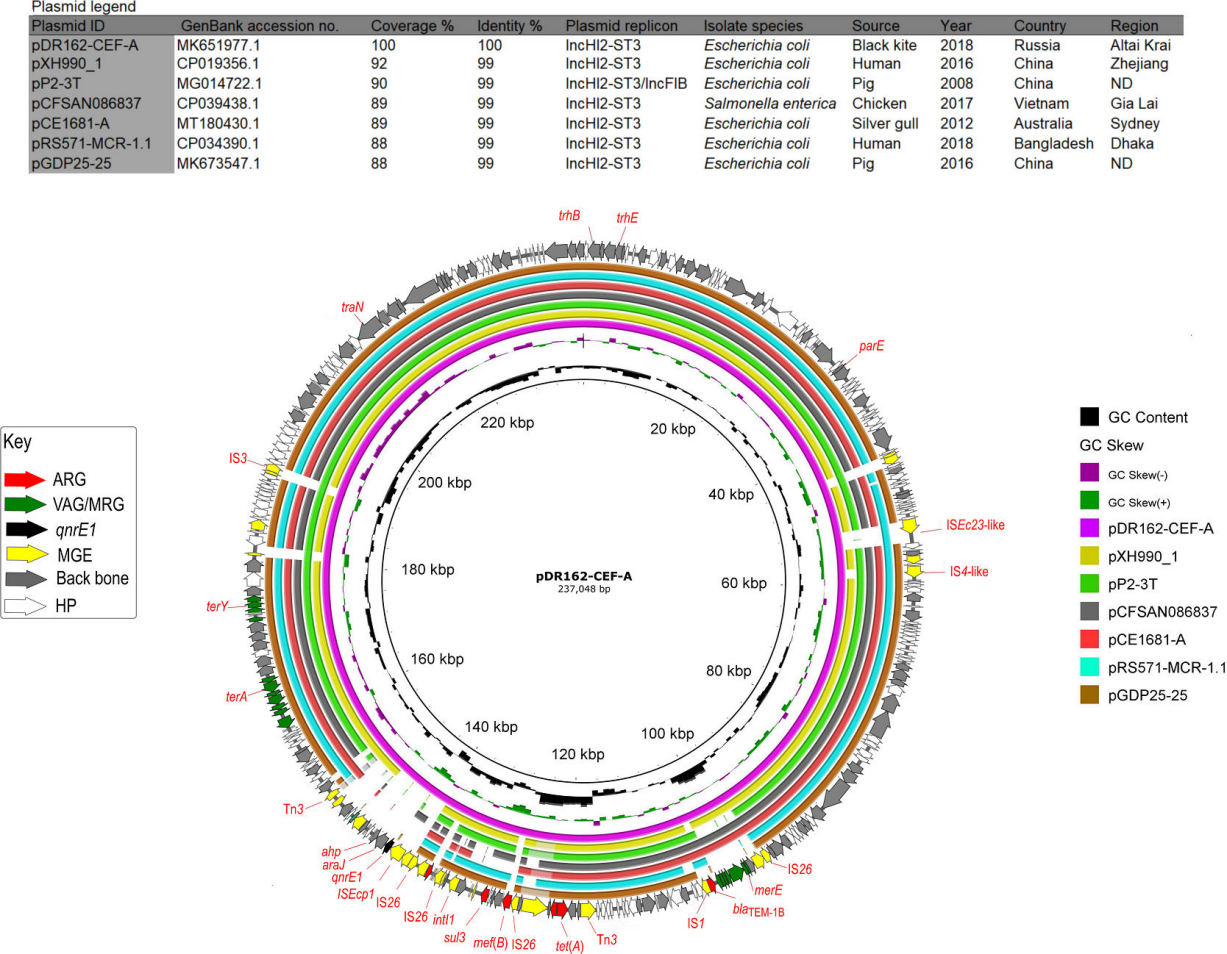
BRIG comparison of *qnrE1*-positive IncHI2-ST3 plasmid pDR162-CEF-A with similar sequences from GenBank. In the key, ARG: antibiotic resistance gene, VAG/MRG: virulence-associated gene/metal resistance gene, MGE: mobile genetic element, and HP: hypothetical protein.

Four complete and closed plasmids were recovered from isolate DR162-CEF of ST354 including pDR162-CEF-A (IncHI2-ST3), pDR162-CEF-B (IncI1-Iα/ST23 of CC2), pDR162-CEF-C (IncFII, replicon ST: F4*:A-:B37*), and pDR162-CEF-D (ColpVC). Plasmid pDR162-CEF-A (237,048 bp) was conjugative and had a typical HI2 backbone structure including regions for replication, stability, maintenance, and horizontal gene transfer (Fig. 4). It harbored five ARGs [*bla*_TEM-1B_, *qnrE1*, *sul3*, *tet*(A), and *dfrA12*] and various metal resistance genes. Most of the ARGs except *bla*_TEM-1B_ were part of a complex region containing *intI1* and an associated resistance region with copies of Tn*3,* IS*26*, IS*Ecp1,* and IS*256*. Similarly to its first report in a *Klebsiella pneumoniae* isolate (26), *qnrE1* was flanked by an IS*Ecp1* upstream and *araj* (transporter of major facilitator superfamily) and a truncated *ahp* (alkyl hydroperoxidase) downstream (Fig. 4). Comparative analysis of pDR162-CEF-A indicated six plasmids sharing similar IncHI2-ST3 backbone structure and tellurium resistance region. They all had antibiotic resistance regions with the absence of IS*Ecp1-qnrE1-araJ*-Δ*ahp* sequence, mostly originated from *E. coli* and were sourced from domestic animals and human sources in various Asian countries (China, Vietnam, and Bangladesh) and from a wildlife sample in Australia (Fig. 4). Notably, three of the compared plasmids (pXH990_1, pRS571-MCR-1.1, and pGDP25-25) carried acquired colistin resistance genes (*mcr-1*), while pCE1781-A hosted a carbapenem resistance gene *bla*_IMP-4_.

Plasmids pDR162-CEF-B (96,414 bp) and pDR162-CEF-C (112,643 bp) carried various genes for colicin activity, colicin immunity, and metal resistance (Fig. S1 and S2). Plasmid pDR162-CEF-B had high coverage and identity with four similar *bla*_CMY-2_-positive IncI1-Iα plasmids from domestic animals (pCVM22462 in *Salmonella enterica* from USA), wildlife (pCE1628_I1 in *E. coli* from Australia), food (pS10584 in *Salmonella enterica* from China), and an unknown source (plasmid_ 2). These plasmids were identical to pDR162-CEF-B except for an insertion of a reverse transcriptase in pDR162-CEF-B and all carried *bla*_CMY-2_ (Fig. S1). Comparative analysis of pDR162-CEF-C identified highly related FII plasmids (pSUO2, p113.6k, and plncF4-A-B1) with different RSTs, all originating from poultry in the USA and sharing plasmid backbone, metal resistance, and VAGs (Fig. S2).

## DISCUSSION

Clinical and agricultural use and misuse of antibiotics are the main drivers for the emergence of antibiotic resistance that can contaminate the ecosystem including the environment and wildlife (45, 46). The existence of *E. coli* in wildlife with multidrug resistance (MDR) to clinically important antibiotics represents a potential hazard to human and animal health (47, 48), where wildlife can act as a reservoir and spreader of ARB in the human-environment-animal interphase (48, 49). Black kites in Russian western Siberia reside in localities with access to human food refuse (i.e., landfills) (50) and habitat intersection with agricultural elements (i.e., fertilized agricultural lands (51) and food animals), all of which are known to be common sources of ARB (52 - 54). These factors support the hypothesis of a potential anthropogenic spillover of ARB and antibiotic-resistant determinants to black kites in the Altai Krai region which warranted this study.

Through this investigation, we identified prominent occurrences of *E. coli* with antibiotic resistance including MDR and ESBL profiles in black kites residing in southwestern Siberia in proximity to human-influenced environments. The high occurrence of antibiotic-resistant *E. coli* in samples near major urban areas such as Biysk and Kyzyl supports the finding of previous studies (55 - 57) associating wildlife in environments influenced by anthropogenic activity with high carriage of ARB. The occurrence of antibiotic-resistant *E. coli* in Kokorya might implicate human populations in rural communities as carriers and possible transmitters of ARB that was observed in studies from rural regions in India (58) and Peru (59). Another possible source for the acquisition and carriage of antibiotic-resistant *E. coli* observed in black kites could be their wintering grounds that are located in the densely populated areas in India and Pakistan where they feed mainly on municipal waste from landfills (13). Considering that sampled kites were in their nestling stage, it is possible that their parents carried the antibiotic-resistant isolates from their wintering habitat in the Indian subcontinent and transmitted observed antibiotic- resistant *E. coli* via feeding to their offspring. Although, it is well known that bird nestlings can get inoculated by different bacteria from their environment particularly food provided by their parents which is mixed with the parents’ saliva (60), reports investigating the carriage duration of bacteria in avian wildlife are still scarce (61, 62).

Our results indicate a high occurrence of antibiotic-resistant (35/55, 63%), MDR (48/51, 94%), and ESBL-producing (18/36, 50%) *E. coli* isolates in black kites in southwestern Siberia. In comparison, studies on wild birds from Europe (3, 63 - 65) show a relatively lower occurrence of antibiotic-resistant *E. coli* and ESBL producers while affirming the spread of MDR phenotype between these isolates. Plaza-Rodriguez et al. (63) identified ESBL-producing *E. coli* in 9.8% (10/102) of samples sourced from wild ducks (subfamily Anatinae) and geese (subfamily Anserinae) in Germany where most of these isolates (70%, 7/10) exhibited MDR profiles. Similarly, Guenther et al. (64) demonstrated the occurrence of ESBL-producing *E. coli* (5.2%, 9/171) in various raptor species in Germany, while a study on wild birds from Portugal (65) identified *E. coli* with ESBL profiles in 26.9% of samples (32/119), all of which were MDR. Skarzynska et al. (3) investigated a collection of samples sourced from free-living wild birds and birds from a rehabilitation center in Poland where they determined a high occurrence of antibiotic-resistant *E. coli* (50%, 35/70), most of which had MDR profiles (77%, 27/35). Several factors hinder proper comparative analysis and interpretation of such monitoring data on antibiotic resistance in wildlife. This includes a difference in study design (selective culturing and WGS versus whole sample metagenomics sequencing), the use of different sampling (cloacal and goiter swabs, fecal droppings, animal tissue) and culturing methods (selective versus nonselective), species and food preference of wild birds and geographic location of sampling.

Black kites in the Biysk region utilize available human food refuse from nearby landfills as their main food source (50). We predict a similar behavior for kites in Kyzyl where a landfill is located in the proximity to the city (Fig. 1). Based on these feeding behaviors, the observed ARB and their resistance profiles in these birds might be associated with anthropogenic spillover that was captured by black kites. This postulation is supported by results from a phylogenetic analysis that identified two clonal *mcr-1*-positive *E. coli* isolates (DR356a-COL and DR358b-COL) originating from two black kites in Kyzyl with zero SNPs difference indicating a recent common source of the two isolates. Notably, these isolates belonged to ST93 which is frequently reported in APEC and ExPEC (66, 67). Similarly, several isolates from kites in Biysk and Kyzyl belonged to STs associated with APEC (ST624 (68)), ExPEC (ST38 (69)), and overlapping APEC-ExPEC (ST10, ST23, ST354, and ST1011 (66, 69, 70)). Besides one isolate of ST162 that is commonly detected in livestock (71, 72), companion animals (73, 74), and wildlife (75, 76), black kites in Kokrya had no isolates with known STs linked to pathogenic *E. coli*. The association of avian wildlife foraging mainly on human food refuse with a significant prevalence of *E. coli* encoding resistance to clinically important antibiotics was well documented in studies involving wild bird species such as gulls (Laridae) (57, 77, 78), white storks (*Ciconia ciconia*) (79), and bald eagles (*Haliaeetus leucocephalus*) (78). These studies reported high diversity of ARGs encoding resistance to multiple classes of antibiotics including ESBL, which was also observed in black kites from Biysk and Kyzyl. The spread of ESBL/AmpC production, chromosomal quinolone resistance and considerable virulence content in sequenced isolates is concerning as it primes them as potentially pathogenic lineages.

Class one integrase gene *intI1* was suggested as a proxy for anthropogenic pollution and as a marker for antibiotic resistance (80). Based on that, the high occurrence of *intI1* in the sequenced isolates from kites in southwestern Siberia (22/36, 61.1%) may indicate a prominent anthropogenic influence on these populations. IS*26* has emerged as a critical element in the mobilization of ARGs in Gram-negative bacteria where it is usually found in complex resistance regions harboring resistance genes to multiple classes of antibiotics (81, 82). The presence of IS*26* in isolates originating from kites in Biysk and Kyzyl might be one of the factors contributing to the high diversity of their ARGs.

Mobile colistin resistance gene *mcr-1* is increasingly observed in avian wildlife including aquatic and migratory species (3, 83). Suggested ability of wild birds for prolonged carriage and transmission of ARB (61) is worrisome especially in the case of migratory birds as black kites harboring isolates with resistance to last-line antibiotics. The spread of identical *mcr-1*-positive IncI2 plasmids in isolates from black kites of different STs and locations might indicate mobilization of the plasmid through horizontal gene transfer or a gradual acquisition of *mcr-1*-positive isolates by black kites on multiple occasions from unknown sources. Identification of highly similar IncI2 plasmids originating from black kites and clinical samples in Russia, China, and Argentina and a sewage sample from China (Fig. 3) supports the reports of global spread of *mcr-1*-positive IncI2 plasmids (84).

The association of DR162-CEF with ExPEC-APEC and its carriage of MDR and virulent plasmids is concerning as *E. coli* ST354 was linked to serious clinical infections including urinary tract, prostate, and bloodstream infections (69, 84, 85).

To our best knowledge, we present the first report of *qnrE1* in *E. coli* from a wildlife source. Recently, quinolone resistance gene *qnrE1* was identified in a *Salmonella enterica* isolate from retail meat in China (86) and in *Enterobacter asburiae* from a clinical sample in Thailand (87). Most reports on *qnrE1* originate from South America where it was found in clinical (commonly on IncM plasmid) and domestic animal samples, hosted by *Klebsiella pneumoniae*, *Salmonella* Typhimurium, and *E. coli* (88 - 93). A recent study indicated a diseased parrot *Amazona aestiva* in Brazil as a carrier of *qnrE1*-positive IncM1 plasmid (94). However, the parrot was a companion animal [confirmed through written confirmation with the corresponding author (94)] which implies a possible human-companion animal transmission. The *qnrE1* gene was also identified in a MDR *K. pneumoniae* isolate from a native Amazonian fish in Brazil where it was harbored in a hybrid IncFIB/IncHI1B plasmid (95). *qnrE1* originated from the chromosome of *Enterobacter* spp. with a suggested role for IS*Ecp1* in its mobilization to *K. pneumoniae* (26). The association of *qnrE1* with IS*Ecp1* and its insertion in an MDR IncHI2-ST3 plasmid (pDR162-CEF-A) in *E. coli* ST354 may indicate an ongoing interspecies dissemination of *qnrE1*, facilitated by IS*Ecp1* and conjugative plasmids. In addition, the association of MDR IncHI2-ST3 plasmids with metal resistance and biocins might facilitate their persistence and spread in the absence of antibiotic selection pressure (96).

There are several limitations in our study including the uneven distribution of samples between black kites in the three sampled locations and the temporal difference in sampling of kites from Biysk (sampled in 2018) and those from Kyzyl and Kokorya (both sampled in 2019). The use of selective cultivation method results in targeting resistant bacterial isolates, thus hindering a better understanding of the avian microflora including common and persistent bacterial lineages. In addition, the unavailability of national clinical and environmental monitoring data on antibiotic resistance in southwestern Siberia prevented a comparative analysis that would potentially support determining possible sources and transmission routes of identified ARB and their associated ARGs.

Our results imply a human factor behind some of the observed diversity in resistance and plasmids profiles, it also demonstrates that even in environments with minimal human activity such as Altai rural communities, *E. coli* with considerable resistance and virulence determinants can be detected. The risk of carriage and transmission of these isolates on domestic animals and humans is not fully understood. We advise further investigation that adopts a One Health approach in investigating ARB in black kites and other avian species to determine the origin, spread, transmission routes, and persistence of these isolates in the human-domestic animal-environment-wildlife interphase. This in turn can help in determining their reservoirs and provide an informative concession of their health risk.

## Data Availability

The sequencing data presented in this study are openly deposited in GenBank within Bioproject PRJNA702622.

## SUPPLEMENTAL MATERIAL

Supplemental Material msphere.00099-23-s0001.docx Figure S1, Figure S2, and legend for Table S1.

Table S1 msphere.00099-23-s0002.xlsx Characteristics of *E. coli* isolates from black kites in southwestern Siberia.

ASM does not own the copyrights to Supplemental Material that may be linked to, or accessed through, an article. The authors have granted ASM a non-exclusive, world-wide license to publish the Supplemental Material files. Please contact the corresponding author directly for reuse.

## References

[1] Arnold KE , Williams NJ , Bennett M . 2016. 'Disperse abroad in the land': the role of wildlife in the dissemination of antimicrobial resistance. Biol Lett 12:20160137. doi:10.1098/rsbl.2016.0137 27531155 PMC5014016

[2] Dolejska M , Literak I . 2019. Wildlife is overlooked in the epidemiology of medically important antibiotic-resistant bacteria. Antimicrob Agents Chemother 63:e01167-19. doi:10.1128/AAC.01167-19 31209001 PMC6658798

[3] Skarżyńska M , Zaja C M , Bomba A , Bocian Ł , Kozdruń W , Polak M , Wia Cek J , Wasyl D . 2021. Antimicrobial resistance glides in the sky-free-living birds as a reservoir of resistant Escherichia coli with zoonotic potential. Front Microbiol 12:656223. doi:10.3389/fmicb.2021.656223 33897669 PMC8062882

[4] Villa L , Guerra B , Schmoger S , Fischer J , Helmuth R , Zong Z , García-Fernández A , Carattoli A . 2015. IncA/C plasmid carrying bla_NDM-1_, bla_CMY-16_, and fosA3 in a Salmonella enterica serovar corvallis strain isolated from a migratory wild bird in Germany. Antimicrob Agents Chemother 59:6597–6600. doi:10.1128/AAC.00944-15 26169417 PMC4576047

[5] Tarabai H , Valcek A , Jamborova I , Vazhov SV , Karyakin IV , Raab R , Literak I , Dolejska M . 2019. Plasmid-mediated mcr-1 colistin resistance in Escherichia coli from a black kite in Russia. Antimicrob Agents Chemother 63:e01266-19. doi:10.1128/AAC.01266-19 31307988 PMC6709458

[6] International B . 2023. Black kite Milvus migrans, on birdlife international. Available from: http://datazone.birdlife.org/species/factsheet/black-kite-milvus-migrans

[7] Karyakin I , Center of Field Studies . 2017. Problem of identification of Eurasian subspecies of the black kite and records of the pariah kite in Southern Siberia, Russia Проблемы Идентификации Евразийских Подвидов Чёрного Коршуна И Встречи Индо-Малайского Подвида В Южной Сибири, Россия. Raptors Conserv, no. 34:49–67. doi:10.19074/1814-8654-2017-34-49-67

[8] Sergio F , Pedrini P , Marchesi L . 2003. Adaptive selection of foraging and nesting habitat by black kites (Milvus migrans) and its implications for conservation: a multi-scale approach. Biol Conserv 112:351–362. doi:10.1016/S0006-3207(02)00332-4

[9] Cortés-Avizanda A , Almaraz P , Carrete M , Sánchez-Zapata JA , Delgado A , Hiraldo F , Donázar JA . 2011. Spatial heterogeneity in resource distribution promotes facultative sociality in two Trans-Saharan migratory birds. PLoS One 6:e21016. doi:10.1371/journal.pone.0021016 21731640 PMC3120827

[10] Andreyenkova NG , Karyakin IV , Starikov IJ , Sauer‐Gürth H , Literák I , Andreyenkov OV , Shnayder EP , Bekmansurov RH , Alexeyenko MN , Wink M , Zhimulev IF . 2021. Phylogeography and demographic history of the black kite Milvus Migrans, a widespread Raptor in Eurasia, Australia and Africa. J Avian Biol 52:e02822. doi:10.1111/jav.02822

[11] Ferguson-Lees J , Christie DA , Franklin K , Burton P , Mead D . 2001. In Raptors of the world. Houghton Mifflin.

[12] Skyrpan M , Panter C , Nachtigall W , Riols R , Systad G , Škrábal J , Literák I . 2021. Kites Milvus migrans lineatus (Milvus migrans migrans/lineatus) are spreading west across Europe. J Ornithol 162:317–323. doi:10.1007/s10336-020-01832-2

[13] Kumar N , Gupta U , Jhala YV , Qureshi Q , Gosler AG , Sergio F . 2020. GPS-telemetry unveils the regular high-elevation crossing of the Himalayas by a migratory raptor: implications for definition of a `` central Asian flyway’’. Sci Rep 10:15988. doi:10.1038/s41598-020-72970-z 32994476 PMC7524735

[14] Literák I , Škrábal J , Karyakin IV , Andreyenkova NG , Vazhov SV . 2022. Black Kites on a flyway between Western Siberia and the Indian Subcontinent. Sci Rep 12:5581. doi:10.1038/s41598-022-09246-1 35368027 PMC8976839

[15] De Giacomo U , Guerrieri G . 2008. The feeding behavior of the black kite (Milvus migrans) in the rubbish dump of Rome. J Raptor Res 42:110–118. doi:10.3356/JRR-07-09.1

[16] Tanferna A , López-Jiménez L , Blas J , Hiraldo F , Sergio F . 2013. Habitat selection by black kite breeders and floaters: implications for conservation management of raptor floaters. Biol Conserv 160:1–9. doi:10.1016/j.biocon.2012.12.031

[17] Makarov DA , Ivanova OE , Karabanov SY , Gergel MA , Pomazkova AV . 2020. Antimicrobial resistance of commensal Escherichia coli from food-producing animals in Russia. Vet World 13:2053–2061. doi:10.14202/vetworld.2020.2053-2061 33281337 PMC7704320

[18] Khmelevtsova LE , Sazykin IS , Azhogina TN , Sazykina MA . 2020. The dissemination of antibiotic resistance in various environmental objects (Russia). Environ Sci Pollut Res Int 27:43569–43581. doi:10.1007/s11356-020-10231-2 32935217

[19] WHO . 2020. Central Asian and European surveillance of antimicrobial resistance: annual report 2020. WHO, Geneva WHO Regional Office for Europe

[20] Kuznetsova MV , Gizatullina JS , Nesterova LY , Starčič Erjavec M . 2020. Escherichia Coli isolated from cases of colibacillosis in Russian poultry farms (Perm Krai): sensitivity to antibiotics and bacteriocins. Microorganisms 8:741. doi:10.3390/microorganisms8050741 32429211 PMC7285186

[21] Rafalskiy V , Pushkar D , Yakovlev S , Epstein O , Putilovskiy M , Tarasov S , Glazunov A , Korenev S , Moiseeva E , Gorelysheva N . 2020. Distribution and antibiotic resistance profile of key gram-negative bacteria that cause community-onset urinary tract infections in the Russian Federation: resource multicentre surveillance 2017 study. J Glob Antimicrob Resist 21:188–194. doi:10.1016/j.jgar.2019.09.008 31525541

[22] Hernandez J , Bonnedahl J , Eliasson I , Wallensten A , Comstedt P , Johansson A , Granholm S , Melhus A , Olsen B , Drobni M . 2010. Globally disseminated human pathogenic Escherichia coli of o25b-st131 clone, harbouring bla_CTX-M-15_, found in Glaucous-winged gull at remote commander Islands, Russia. Environ Microbiol Rep 2:329–332. doi:10.1111/j.1758-2229.2010.00142.x 23766085

[23] Nordmann P , Jayol A , Poirel L . 2016. A universal culture medium for screening polymyxin-resistant gram-negative isolates. J Clin Microbiol 54:1395–1399. doi:10.1128/JCM.00446-16 26984971 PMC4844728

[24] Strejcek M , Smrhova T , Junkova P , Uhlik O . 2018. Whole-Cell MALDI-TOF MS versus 16S rRNA gene analysis for identification and dereplication of recurrent bacterial isolates. Front Microbiol 9:1294. doi:10.3389/fmicb.2018.01294 29971049 PMC6018384

[25] Kutilova I , Medvecky M , Leekitcharoenphon P , Munk P , Masarikova M , Davidova-Gerzova L , Jamborova I , Bortolaia V , Pamp SJ , Dolejska M . 2021. Extended-Spectrum beta-lactamase-producing Escherichia coli and antimicrobial resistance in municipal and hospital wastewaters in Czech Republic: culture-based and metagenomic approaches. Environ Res 193:110487. doi:10.1016/j.envres.2020.110487 33232750

[26] Albornoz E , Tijet N , De Belder D , Gomez S , Martino F , Corso A , Melano RG , Petroni A . 2017. QnrE1, a member of a new family of plasmid-located quinolone resistance genes, originated from the chromosome of Enterobacter species. Antimicrob Agents Chemother 61:e02555-16. doi:10.1128/AAC.02555-16 28193666 PMC5404601

[27] Dobiasova H , Dolejska M , Jamborova I , Brhelova E , Blazkova L , Papousek I , Kozlova M , Klimes J , Cizek A , Literak I . 2013. Extended spectrum beta-lactamase and fluoroquinolone resistance genes and plasmids among Escherichia coli isolates from zoo animals, Czech Republic. FEMS Microbiol Ecol 85:604–611. doi:10.1111/1574-6941.12149 23679004

[28] Rebelo AR , Bortolaia V , Kjeldgaard JS , Pedersen SK , Leekitcharoenphon P , Hansen IM , Guerra B , Malorny B , Borowiak M , Hammerl JA , Battisti A , Franco A , Alba P , Perrin-Guyomard A , Granier SA , De Frutos Escobar C , Malhotra-Kumar S , Villa L , Carattoli A , Hendriksen RS . 2018. Multiplex PCR for detection of plasmid-mediated colistin resistance determinants, mcr-1, mcr-2, mcr-3, mcr-4 and mcr-5 for surveillance purposes. Euro Surveill 23:17-00672. doi:10.2807/1560-7917.ES.2018.23.6.17-00672 PMC5824125 29439754

[29] EUCAST . 2019. In Antimicrobial susceptibility testing, EUCAST disk diffusion method

[30] EUCAST . 2019. In Breakpoint tables for interpretation of MICs and zone diameters

[31] CLSI . 2017. In Performance standards for antimicrobial susceptibility testing

[32] Cusack TP , Ashley EA , Ling CL , Roberts T , Turner P , Wangrangsimakul T , Dance DAB . 2019. Time to switch from CLSI to EUCAST? A Southeast Asian perspective. Clin Microbiol Infect 25:782–785. doi:10.1016/j.cmi.2019.03.016 30922928 PMC6587905

[33] Kahlmeter G , Giske CG , Kirn TJ , Sharp SE . 2019. Point-counterpoint: differences between the European Committee on antimicrobial susceptibility testing and clinical and laboratory standards Institute recommendations for reporting antimicrobial susceptibility results. J Clin Microbiol 57:e01129-19. doi:10.1128/JCM.01129-19 31315957 PMC6711922

[34] Jouy E , Haenni M , Le Devendec L , Le Roux A , Châtre P , Madec J-Y , Kempf I . 2017. Improvement in routine detection of colistin resistance in E. coli isolated in veterinary diagnostic laboratories. J Microbiol Methods 132:125–127. doi:10.1016/j.mimet.2016.11.017 27894831

[35] Bolger AM , Lohse M , Usadel B . 2014. Trimmomatic: a flexible trimmer for Illumina sequence data. Bioinformatics 30:2114–2120. doi:10.1093/bioinformatics/btu170 24695404 PMC4103590

[36] Bankevich A , Nurk S , Antipov D , Gurevich AA , Dvorkin M , Kulikov AS , Lesin VM , Nikolenko SI , Pham S , Prjibelski AD , Pyshkin AV , Sirotkin AV , Vyahhi N , Tesler G , Alekseyev MA , Pevzner PA . 2012. SPAdes: a new genome assembly algorithm and its applications to single-cell sequencing. J Comput Biol 19:455–477. doi:10.1089/cmb.2012.0021 22506599 PMC3342519

[37] Chen L , Zheng D , Liu B , Yang J , Jin Q . 2016. VFDB 2016: hierarchical and refined dataset for big data analysis -- 10 years on. Nucleic Acids Res 44:D694–D697. doi:10.1093/nar/gkv1239 26578559 PMC4702877

[38] Brettin T , Davis JJ , Disz T , Edwards RA , Gerdes S , Olsen GJ , Olson R , Overbeek R , Parrello B , Pusch GD , Shukla M , Thomason JA , Stevens R , Vonstein V , Wattam AR , Xia F . 2015. RASTtk: a modular and extensible implementation of the RAST algorithm for building custom annotation pipelines and annotating batches of genomes. Sci Rep 5:8365. doi:10.1038/srep08365 25666585 PMC4322359

[39] Alikhan N-F , Petty NK , Ben Zakour NL , Beatson SA . 2011. BLAST ring image generator (BRIG): simple prokaryote genome comparisons. BMC Genomics 12:402. doi:10.1186/1471-2164-12-402 21824423 PMC3163573

[40] Kaas RS , Leekitcharoenphon P , Aarestrup FM , Lund O . 2014. Solving the problem of comparing whole bacterial genomes across different sequencing platforms. PLoS One 9:e104984. doi:10.1371/journal.pone.0104984 25110940 PMC4128722

[41] Letunic I , Bork P . 2021. Interactive tree of life (iTOL) V5: an online tool for phylogenetic tree display and annotation. Nucleic Acids Res 49:W293–W296. doi:10.1093/nar/gkab301 33885785 PMC8265157

[42] Valcek A , Overballe-Petersen S , Hansen F , Dolejska M , Hasman H . 2019. Complete genome sequence of Escherichia coli MT102, a plasmid-free recipient resistant to rifampin, azide, and streptomycin, used in conjugation experiments. Microbiol Resour Announc 8:e00383-19. doi:10.1128/MRA.00383-19 31097507 PMC6522792

[43] Jamborova I , Dolejska M , Vojtech J , Guenther S , Uricariu R , Drozdowska J , Papousek I , Pasekova K , Meissner W , Hordowski J , Cizek A , Literak I . 2015. Plasmid-Mediated resistance to cephalosporins and fluoroquinolones in various Escherichia coli sequence types isolated from rooks wintering in Europe. Appl Environ Microbiol 81:648–657. doi:10.1128/AEM.02459-14 25381245 PMC4277596

[44] Carattoli A , Bertini A , Villa L , Falbo V , Hopkins KL , Threlfall EJ . 2005. Identification of plasmids by PCR-based replicon typing. J Microbiol Methods 63:219–228. doi:10.1016/j.mimet.2005.03.018 15935499

[45] Manyi-Loh C , Mamphweli S , Meyer E , Okoh A . 2018. Antibiotic use in agriculture and its consequential resistance in environmental sources: potential public health implications. Molecules 23:795. doi:10.3390/molecules23040795 29601469 PMC6017557

[46] Larsson DGJ , Flach C-F . 2022. Antibiotic resistance in the environment. Nat Rev Microbiol 20:257–269. doi:10.1038/s41579-021-00649-x 34737424 PMC8567979

[47] Greig J , Rajić A , Young I , Mascarenhas M , Waddell L , LeJeune J . 2015. A scoping review of the role of wildlife in the transmission of bacterial pathogens and antimicrobial resistance to the food chain. Zoonoses Public Health 62:269–284. doi:10.1111/zph.12147 25175882

[48] Lee S , Fan P , Liu T , Yang A , Boughton RK , Pepin KM , Miller RS , Jeong KC . 2022. Transmission of antibiotic resistance at the wildlife-livestock interface. Commun Biol 5:585. doi:10.1038/s42003-022-03520-8 35705693 PMC9200806

[49] Radhouani H , Silva N , Poeta P , Torres C , Correia S , Igrejas G . 2014. Potential impact of antimicrobial resistance in wildlife, environment and human health. Front Microbiol 5:23. doi:10.3389/fmicb.2014.00023 24550896 PMC3913889

[50] Bachtin R , Vazhov S , Makarov AJRC . 2010. In Ecology of synanthropic populations of the black kite in the vicinities of biysk. Altai Kray, Russia.

[51] Ponkina E , Bavorova M , prishepov A , Kovaleva I . 2013. Positive quantitative analysis of farms in Altai Krai, Russia regarding natural conditions, structure, production program, factor endowment, productivity, economic success and income. Barnaul Altai State Agrarian University of Barnaul Subproject No. 8. doi:10.13140/RG.2.1.3702.1202

[52] Haulisah NA , Hassan L , Bejo SK , Jajere SM , Ahmad NI . 2021. High levels of antibiotic resistance in isolates from diseased livestock. Front Vet Sci 8:652351. doi:10.3389/fvets.2021.652351 33869326 PMC8047425

[53] Zhang R , Yang S , An Y , Wang Y , Lei Y , Song L . 2022. Antibiotics and antibiotic resistance genes in landfills: a review. Sci Total Environ 806:150647. doi:10.1016/j.scitotenv.2021.150647 34597560

[54] Jauregi L , Epelde L , Alkorta I , Garbisu C . 2021. Antibiotic resistance in agricultural soil and crops associated to the application of cow manure-derived amendments from conventional and organic livestock farms. Front Vet Sci 8:633858. doi:10.3389/fvets.2021.633858 33708812 PMC7940349

[55] Atterby C , Ramey AM , Hall GG , Järhult J , Börjesson S , Bonnedahl J . 2016. Increased prevalence of antibiotic-resistant E. coli in gulls sampled in southcentral Alaska is associated with urban environments. Infect Ecol Epidemiol 6:32334. doi:10.3402/iee.v6.32334 27649798 PMC5030259

[56] Jarma D , Sánchez MI , Green AJ , Peralta-Sánchez JM , Hortas F , Sánchez-Melsió A , Borrego CM . 2021. Faecal microbiota and antibiotic resistance genes in migratory waterbirds with contrasting habitat use. Sci Total Environ 783:146872. doi:10.1016/j.scitotenv.2021.146872 33872913

[57] Wyrsch ER , Nesporova K , Tarabai H , Jamborova I , Bitar I , Literak I , Dolejska M , Djordjevic SP . 2022. Urban wildlife crisis: Australian silver gull is a bystander host to widespread clinical antibiotic resistance. mSystems 7:e0015822. doi:10.1128/msystems.00158-22 35469421 PMC9238384

[58] Purohit MR , Chandran S , Shah H , Diwan V , Tamhankar AJ , Stålsby Lundborg C . 2017. Antibiotic resistance in an Indian rural community: a 'one-health' observational study on commensal coliform from humans, animals, and water. Int J Environ Res Public Health 14:386. doi:10.3390/ijerph14040386 28383517 PMC5409587

[59] Bartoloni A , Pallecchi L , Rodríguez H , Fernandez C , Mantella A , Bartalesi F , Strohmeyer M , Kristiansson C , Gotuzzo E , Paradisi F , Rossolini GM . 2009. Antibiotic resistance in a very remote amazonas community. Int J Antimicrob Agents 33:125–129. doi:10.1016/j.ijantimicag.2008.07.029 18947984

[60] Lombardo MP , Thorpe PA , Cichewicz R , Henshaw M , Millard C , Steen C , Zeller TK . 1996. Communities of cloacal bacteria in tree swallow families. The Condor 98:167–172. doi:10.2307/1369521

[61] Sandegren L , Stedt J , Lustig U , Bonnedahl J , Andersson DI , Järhult JD . 2018. Long-Term carriage and rapid transmission of extended spectrum beta-lactamase-producing E. coli within a flock of mallards in the absence of antibiotic selection. Environ Microbiol Rep 10:576–582. doi:10.1111/1758-2229.12681 30043488

[62] Franklin AB , Ramey AM , Bentler KT , Barrett NL , McCurdy LM , Ahlstrom CA , Bonnedahl J , Shriner SA , Chandler JC . 2020. Gulls as sources of environmental contamination by colistin-resistant bacteria. Sci Rep 10:4408. doi:10.1038/s41598-020-61318-2 32157139 PMC7064522

[63] Plaza-Rodríguez C , Alt K , Grobbel M , Hammerl JA , Irrgang A , Szabo I , Stingl K , Schuh E , Wiehle L , Pfefferkorn B , Naumann S , Kaesbohrer A , Tenhagen B-A . 2020. Wildlife as sentinels of antimicrobial resistance in Germany? Front Vet Sci 7:627821. doi:10.3389/fvets.2020.627821 33585611 PMC7873465

[64] Guenther S , Aschenbrenner K , Stamm I , Bethe A , Semmler T , Stubbe A , Stubbe M , Batsajkhan N , Glupczynski Y , Wieler LH , Ewers C . 2012. Comparable high rates of extended-spectrum-beta-lactamase-producing Escherichia coli in birds of prey from Germany and Mongolia. PLoS One 7:e53039. doi:10.1371/journal.pone.0053039 23300857 PMC3534101

[65] Pinto L , Radhouani H , Coelho C , Martins da Costa P , Simões R , Brandão RML , Torres C , Igrejas G , Poeta P . 2010. Genetic detection of extended-spectrum beta-lactamase-containing Escherichia coli isolates from birds of prey from serra da Estrela natural reserve in Portugal. Appl Environ Microbiol 76:4118–4120. doi:10.1128/AEM.02761-09 20418435 PMC2893492

[66] Maluta RP , Logue CM , Casas MRT , Meng T , Guastalli EAL , Rojas TCG , Montelli AC , Sadatsune T , de Carvalho Ramos M , Nolan LK , da Silveira WD . 2014. Overlapped sequence types (STs) and serogroups of avian pathogenic (APEC) and human extra-intestinal pathogenic (ExPEC) Escherichia coli isolated in Brazil. PLoS One 9:e105016. doi:10.1371/journal.pone.0105016 25115913 PMC4130637

[67] Lu Q , Zhang W , Luo L , Wang H , Shao H , Zhang T , Luo Q . 2022. Genetic diversity and multidrug resistance of phylogenic groups B2 and D in inpec and expec isolated from chickens in central China. BMC Microbiol 22:60. doi:10.1186/s12866-022-02469-2 35180845 PMC8855568

[68] Solà-Ginés M , Cameron-Veas K , Badiola I , Dolz R , Majó N , Dahbi G , Viso S , Mora A , Blanco J , Piedra-Carrasco N , González-López JJ , Migura-Garcia L . 2015. Diversity of multi-drug resistant avian pathogenic Escherichia coli (APEC) causing outbreaks of colibacillosis in broilers during 2012 in Spain. PLoS One 10:e0143191. doi:10.1371/journal.pone.0143191 26600205 PMC4657910

[69] Manges AR , Geum HM , Guo A , Edens TJ , Fibke CD , Pitout JDD . 2019. Global extraintestinal pathogenic Escherichia coli (ExPEC) lineages. Clin Microbiol Rev 32:e00135-18. doi:10.1128/CMR.00135-18 31189557 PMC6589867

[70] Mehat JW , van Vliet AHM , La Ragione RM . 2021. The avian pathogenic Escherichia coli (APEC) pathotype is comprised of multiple distinct, independent genotypes. Avian Pathol 50:402–416. doi:10.1080/03079457.2021.1915960 34047644

[71] Dahms C , Hübner N-O , Kossow A , Mellmann A , Dittmann K , Kramer A . 2015. Occurrence of ESBL-producing Escherichia coli in livestock and farm workers in Mecklenburg-Western Pomerania, Germany. PLoS One 10:e0143326. doi:10.1371/journal.pone.0143326 26606146 PMC4659621

[72] Umpiérrez A , Bado I , Oliver M , Acquistapace S , Etcheverría A , Padola NL , Vignoli R , Zunino P . 2017. Zoonotic potential and antibiotic resistance of Escherichia coli in neonatal calves in Uruguay. Microbes Environ 32:275–282. doi:10.1264/jsme2.ME17046 28904264 PMC5606698

[73] Moon DC , Mechesso AF , Kang HY , Kim S-J , Choi J-H , Kim MH , Song H-J , Yoon S-S , Lim S-K . 2020. First report of an Escherichia Coli strain carrying the colistin resistance determinant mcr-1 from a dog in South Korea. Antibiotics (Basel) 9:768. doi:10.3390/antibiotics9110768 33147688 PMC7694106

[74] Dierikx CM , van Duijkeren E , Schoormans AHW , van Essen-Zandbergen A , Veldman K , Kant A , Huijsdens XW , van der Zwaluw K , Wagenaar JA , Mevius DJ . 2012. Occurrence and characteristics of extended-spectrum-β-lactamase- and ampc-producing clinical isolates derived from companion animals and horses. J Antimicrob Chemother 67:1368–1374. doi:10.1093/jac/dks049 22382469

[75] Wyrsch ER , Chowdhury PR , Wallis L , Cummins ML , Zingali T , Brandis KJ , Djordjevic SP . 2020. Whole-genome sequence analysis of environmental Escherichia Coli from the faeces of straw-Necked Ibis (Threskiornis spinicollis) nesting on inland wetlands. Microb Genom 6:e000385. doi:10.1099/mgen.0.000385 32519939 PMC7371105

[76] Fuentes-Castillo D , Esposito F , Cardoso B , Dalazen G , Moura Q , Fuga B , Fontana H , Cerdeira L , Dropa M , Rottmann J , González-Acuña D , Catão-Dias JL , Lincopan N . 2020. Genomic data reveal international lineages of critical priority Escherichia coli harbouring wide resistome in Andean condors (vultur gryphus Linnaeus, 1758). Mol Ecol 29:1919–1935. doi:10.1111/mec.15455 32335957

[77] Mukerji S , Gunasekera S , Dunlop JN , Stegger M , Jordan D , Laird T , Abraham RJ , Barton M , O’Dea M , Abraham S . 2020. Implications of foraging and interspecies interactions of birds for carriage of Escherichia coli strains resistant to critically important antimicrobials. Appl Environ Microbiol 86:e01610-20. doi:10.1128/AEM.01610-20 32801178 PMC7531969

[78] Ahlstrom CA , Bonnedahl J , Woksepp H , Hernandez J , Olsen B , Ramey AM . 2018. Acquisition and dissemination of cephalosporin-resistant E. coli in migratory birds sampled at an Alaska landfill as inferred through genomic analysis. Sci Rep 8:7361. doi:10.1038/s41598-018-25474-w 29743625 PMC5943298

[79] Höfle U , Jose Gonzalez-Lopez J , Camacho MC , Solà-Ginés M , Moreno-Mingorance A , Manuel Hernández J , De La Puente J , Pineda-Pampliega J , Aguirre JI , Torres-Medina F , Ramis A , Majó N , Blas J , Migura-Garcia L . 2020. Foraging at solid urban waste disposal sites as risk factor for cephalosporin and colistin resistant Escherichia Coli carriage in white storks (Ciconia ciconia). Front Microbiol 11:1397. doi:10.3389/fmicb.2020.01397 32849315 PMC7399022

[80] Roe MT , Vega E , Pillai SD . 2003. Antimicrobial resistance markers of class 1 and class 2 integron-bearing Escherichia coli from irrigation water and sediments. Emerg Infect Dis 9:822–826. doi:10.3201/eid0907.020529 12890322 PMC3023436

[81] Harmer CJ , Moran RA , Hall RM . 2014. Movement of IS26-associated antibiotic resistance genes occurs via a translocatable unit that includes a single IS26 and preferentially inserts adjacent to another IS26. mBio 5:e01801–14. doi:10.1128/mBio.01801-14 25293759 PMC4196232

[82] Varani A , He S , Siguier P , Ross K , Chandler M . 2021. The Is6 family, a clinically important group of insertion sequences including IS26. Mob DNA 12:11. doi:10.1186/s13100-021-00239-x 33757578 PMC7986276

[83] Ruzauskas M , Vaskeviciute L . 2016. Detection of the mcr-1 gene in Escherichia coli prevalent in the migratory bird species Larus argentatus. J Antimicrob Chemother 71:2333–2334. doi:10.1093/jac/dkw245 27330066

[84] Guo S , Wakeham D , Brouwers HJM , Cobbold RN , Abraham S , Mollinger JL , Johnson JR , Chapman TA , Gordon DM , Barrs VR , Trott DJ . 2015. Human-Associated fluoroquinolone-resistant Escherichia coli clonal lineages, including ST354, isolated from canine feces and extraintestinal infections in Australia. Microbes Infect 17:266–274. doi:10.1016/j.micinf.2014.12.016 25576024

[85] Mora A , Blanco M , López C , Mamani R , Blanco JE , Alonso MP , García-Garrote F , Dahbi G , Herrera A , Fernández A , Fernández B , Agulla A , Bou G , Blanco J . 2011. Emergence of clonal groups O1:HNM-D-ST59, O15:H1-D-ST393, O20:H34/HNM-D-ST354, O25b:H4-B2-ST131 and ONT:H21,42-B1-ST101 among CTX-M-14-producing Escherichia coli clinical isolates in Galicia, northwest Spain. Int J Antimicrob Agents 37:16–21. doi:10.1016/j.ijantimicag.2010.09.012 21075606

[86] Lyu N , Feng Y , Pan Y , Huang H , Liu Y , Xue C , Zhu B , Hu Y . 2021. Genomic characterization of Salmonella enterica isolates from retail meat in Beijing, China. Front Microbiol 12:636332. doi:10.3389/fmicb.2021.636332 33897640 PMC8058101

[87] Kerdsin A , Deekae S , Chayangsu S , Hatrongjit R , Chopjitt P , Takeuchi D , Akeda Y , Tomono K , Hamada S . 2019. Genomic characterization of an emerging bla_KPC-2_ carrying Enterobacteriaceae clinical isolates in Thailand. Sci Rep 9:18521. doi:10.1038/s41598-019-55008-x 31811215 PMC6898716

[88] Coppola N , Freire B , Umpiérrez A , Cordeiro NF , Ávila P , Trenchi G , Castro G , Casaux ML , Fraga M , Zunino P , Bado I , Vignoli R . 2020. Transferable resistance to highest priority critically important antibiotics for human health in Escherichia Coli strains obtained from livestock Feces in Uruguay. Front Vet Sci 7:588919. doi:10.3389/fvets.2020.588919 33330715 PMC7717973

[89] Coppola N , Cordeiro NF , Trenchi G , Esposito F , Fuga B , Fuentes-Castillo D , Lincopan N , Iriarte A , Bado I , Vignoli R . 2022. Imported one-day-old chicks as Trojan horses for multidrug-resistant priority pathogens harboring mcr-9, rmtG, and extended-spectrum beta-lactamase genes. Appl Environ Microbiol 88:e0167521. doi:10.1128/AEM.01675-21 34731047 PMC8788672

[90] Calarga AP , Gontijo MTP , de Almeida LGP , de Vasconcelos ATR , Nascimento LC , de Moraes Barbosa TMC , de Carvalho Perri TM , Dos Santos SR , Tiba-Casas MR , Marques EGL , Ferreira CM , Brocchi M . 2022. Antimicrobial resistance and genetic background of non-typhoidal Salmonella enterica strains isolated from human infections in São Paulo, Brazil (2000-2019). Braz J Microbiol 53:1249–1262. doi:10.1007/s42770-022-00748-8 35446010 PMC9433476

[91] Sartori L , Sellera FP , Moura Q , Cardoso B , Fontana H , Côrtes LA , Cerdeira L , Lincopan N . 2020. Genomic features of a polymyxin-resistant Klebsiella pneumoniae ST491 isolate co-harbouring bla_CTX-M-8_ and qnrE1 genes from a hospitalised cat in São Paulo, Brazil. J Glob Antimicrob Resist 21:186–187. doi:10.1016/j.jgar.2020.03.006 32224265

[92] Soares FB , Camargo CH , Cunha MPV , de Almeida EA , Bertani AM de J , Carvalho E de , de Paiva JB , Fernandes SA , Tiba-Casas MR . 2019. Co-Occurrence of qnre1 and bla_CTX-M-8_ in incm1 transferable plasmids contributing to MDR in different Salmonella serotypes. J Antimicrob Chemother 74:1155–1156. doi:10.1093/jac/dky516 30541085

[93] Monte DF , Lincopan N , Cerdeira L , Fedorka-Cray PJ , Landgraf M . 2019. Early dissemination of qnrE1 in salmonella enterica serovar typhimurium from livestock in South America. Antimicrob Agents Chemother 63:e00571-19. doi:10.1128/AAC.00571-19 31235627 PMC6709493

[94] Cunha MPV , Davies YM , Cerdeira L , Dropa M , Lincopan N , Knöbl T . 2017. Complete DNA sequence of an IncM1 plasmid bearing the novel qnrE1 plasmid-mediated quinolone resistance variant and bla_CTX-M-8_ from klebsiella pneumoniae sequence type 147. Antimicrob Agents Chemother 61:e00592-17. doi:10.1128/AAC.00592-17 28652231 PMC5571290

[95] Cerdeira L , Monte DFM , Fuga B , Sellera FP , Neves I , Rodrigues L , Landgraf M , Lincopan N . 2020. Genomic insights of Klebsiella pneumoniae isolated from a native Amazonian fish reveal wide resistome against heavy metals, disinfectants, and clinically relevant antibiotics. Genomics 112:5143–5146. doi:10.1016/j.ygeno.2020.09.015 32916256 PMC7758709

[96] Wyrsch ER , Reid CJ , DeMaere MZ , Liu MY , Chapman TA , Roy Chowdhury P , Djordjevic SP . 2019. Complete sequences of multiple-drug resistant IncHI2 ST3 plasmids in Escherichia coli of porcine origin in Australia. Front Sustain Food Syst 3. doi:10.3389/fsufs.2019.00018

