# Supplementary material for: Clinically relevant antibiotic resistance in Escherichia coli from black kites in southwestern Siberia: a genetic and phenotypic investigation
Source: mSphere. 2023 Jun 13;8(4):e00099-23. doi: 10.1128/msphere.00099-23 (PMC10449506; doi:10.1128/msphere.00099-23)
Supplement: Supplemental Material — Figure S1, Figure S2, and legend for Table S1. [file msphere.00099-23-s0001.docx]

**Supplementary Figure S1**

**
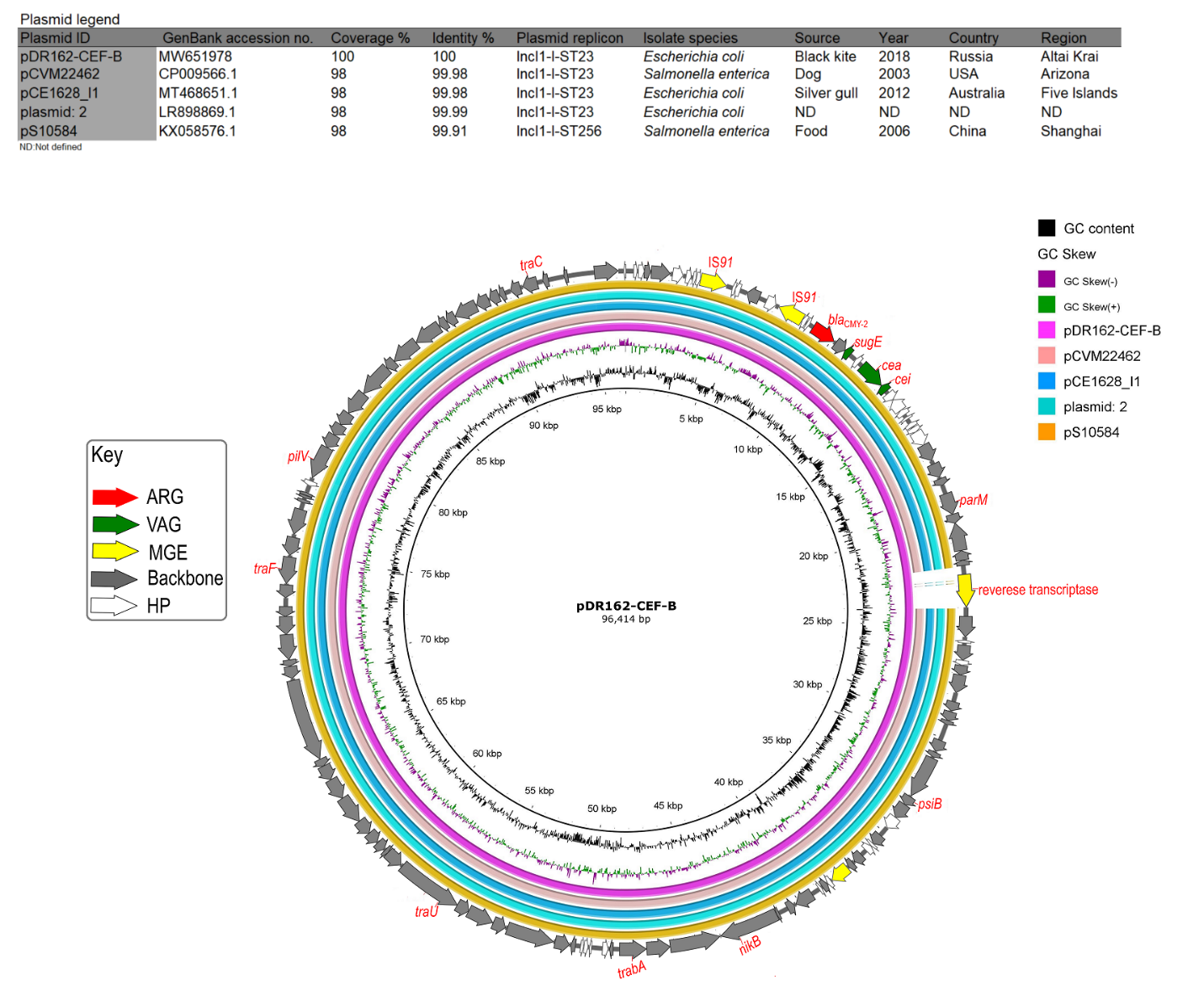
**

**Supplementary Figure S1.** BRIG comparison of IncI1-I ST23 plasmid pDR162-CEF-B with similar sequences from GenBank. In the key, ARG: antibiotic resistance gene, VAG: virulence-associated gene, MGE: mobile genetic element and HP: hypothetical protein.

**Supplementary Figure S2**


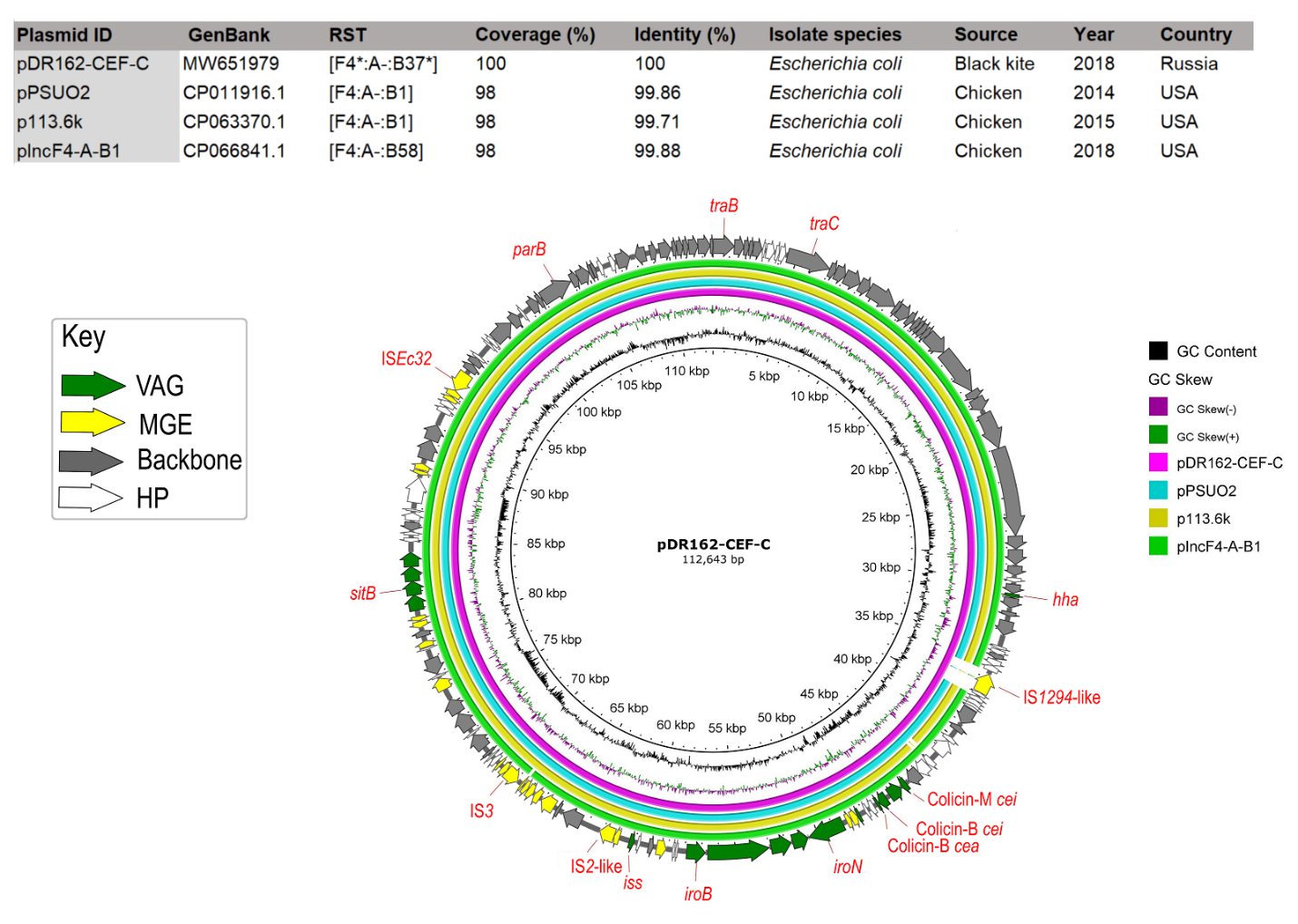


**Supplementary Figure S2.** BRIG comparison of virulent IncFII plasmid pDR162-CEF-C with similar sequences from GenBank. In the key, VAG: virulence-associated gene, MGE: mobile genetic element and HP: hypothetical protein.

**Supplementary Table S1.** Characteristics of *E. coli* isolates from black kites in southwestern Siberia. Localities L1: Biysk, L2: Kyzyl and L3: Kokorya. Boxes highlighted in yellow and + sign indicate the presence of gene, plasmid or multi-drug resistant isolates while empty boxes indicate the absence of gene, plasmid or MDR isolates. In antibiotic susceptibility testing, boxes include the inhibition diameter (mm.) of tested antibiotic by disk diffusion method (AMP: ampicillin, S: streptomycin, S3: sulfonamides, TE: tetracycline, SXT: trimethoprim/sulfamethoxazole, C: chloramphenicol, KZ: cefazolin, NA: nalidixic acid, CAZ: ceftazidime, CN: gentamicin, AMC: amoxicillin/clavulanic acid, CIP: ciprofloxacin, FOS: Fosfomycin, ETP: etrapenem, IPM: imipenem, ATM: aztreonam, F: nitrofurantoin, AZM: azithromycin, and CT: colistin). Colors of boxes are used as follows: complete resistance, orange; intermediate resistance, blue; susceptibility, white. In MAST disk test, NEG: no AmpC, ESBL or suspected carbapenemase production, ESBL: Extended-spectrum beta-lactamase producer and AmpC: AmpC beta-lactamase producer. In plasmid replicon sequence type, * indicate the closest sequence type and CC: clonal complex. In selection media, CEF 2.0: cefotaxime 2.0 mg/L, CIP 0.5: ciprofloxacin 0.05 mg/L, COL 3.0: colistin 3 mg/L. In whole genome sequence (WGS), isolates that undergone WGS are highlighted in green with + sign. Other abbreviations: RU: Russia, BK: black kite, ST: sequence type.
